# A case study on decentralized manufacturing of 3D printed medicines

**DOI:** 10.1016/j.ijpx.2023.100184

**Published:** 2023-05-30

**Authors:** Iria Seoane-Viaño, Xiaoyan Xu, Jun Jie Ong, Ahmed Teyeb, Simon Gaisford, André Campos-Álvarez, Anja Stulz, Carmen Marcuta, Lilia Kraschew, Wolfgang Mohr, Abdul W. Basit, Alvaro Goyanes

**Affiliations:** a Department of Pharmaceutics, UCL School of Pharmacy, University College London, 29-39 Brunswick Square, London WC1N 1AX, UK; b Department of Pharmacology, Pharmacy and Pharmaceutical Technology, Paraquasil Group (GI-2109), Faculty of Pharmacy, iMATUS and Health Research Institute of Santiago de Compostela (IDIS), University of Santiago de Compostela (USC), Santiago de Compostela 15782, Spain; c Brunel Innovation Centre, Brunel University London, Uxbridge UB8 3PH, UK; d FABRX Artificial Intelligence, Carretera de Escairón, 14, Currelos (O Saviñao), CP 27543, Spain; e FABRX Ltd., Henwood House, Henwood, Ashford TN24 8DH, UK; f Losan Pharma GmbH, Otto-Hahn-Strasse 13, 79395 Neuenburg, Germany; g Departamento de Farmacología, Farmacia y Tecnología Farmacéutica, I+D Farma Group (GI-1645), Facultad de Farmacia, iMATUS and Health Research Institute of Santiago de Compostela (IDIS), Universidade de Santiago de Compostela (USC), Santiago de Compostela 15782, Spain

**Keywords:** Real-time release testing, Direct powder extrusion of personalized pharmaceuticals, Process analytical technologies, Decentralised and distributed fabrication of formulations, Additive manufacturing of drug products and drug delivery systems, Digital healthcare and industry 4.0, Three-dimensional printing using M3DIMAKER

## Abstract

Pharmaceutical 3D printing (3DP) is one of the emerging enabling technologies of personalised medicines as it affords the ability to fabricate highly versatile dosage forms. In the past 2 years, national medicines regulatory authorities have held consultations with external stakeholders to adapt regulatory frameworks to embrace point-of-care manufacturing. The proposed concept of decentralized manufacturing (DM) involves the provision of feedstock intermediates (pharma-inks) prepared by pharmaceutical companies to DM sites for manufacturing into the final medicine. In this study, we examine the feasibility of this model, with respect to both manufacturing and quality control. Efavirenz-loaded granulates (0–35%*w*/w) were produced by a manufacturing partner and shipped to a 3DP site in a different country. Direct powder extrusion (DPE) 3DP was subsequently used to prepare printlets (3D printed tablets), with mass ranging 266–371 mg. All printlets released more than 80% drug load within the first 60 min of the in vitro drug release test. An in-line near-infrared spectroscopy system was used as a process analytical technology (PAT) to quantify the printlets' drug load. Calibration models were developed using partial least squares regression, which showed excellent linearity (R^2^ = 0.9833) and accuracy (RMSE = 1.0662). Overall, this work is the first to report the use of an in-line NIR system to perform real-time analysis of printlets prepared using pharma-inks produced by a pharmaceutical company. By demonstrating the feasibility of the proposed distribution model through this proof-of-concept study, this work paves the way for investigation of further PAT tools for quality control in 3DP point-of-care manufacturing.

## Graphical abstract

**Figure:**
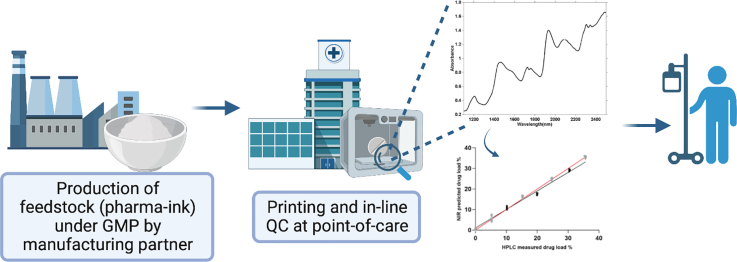

## 1 Introduction

Three-dimensional (3D) printing possesses the potential to bring personalised medicine closer to the patient by enabling drug manufacturing at the point-of-care (Beer et al., 2021; Fastø et al., 2019; Funk et al., 2022; Goyanes et al., 2019; Lu et al., 2022; Patel et al., 2021; Seoane-Viaño et al., 2021a). Small batches of printlets can be prepared on-demand with tailored drug doses, allowing bespoke pharmaceutical treatments to be delivered based on each patient's unique physiological and genetic makeup (Andreadis et al., 2022; Prendergast and Burdick, 2020; Rodríguez-Pombo et al., 2023; Windolf et al., 2022). Almost a decade of excitement around the disruptive potential of pharmaceutical 3D printing has recently cumulated in regulatory bodies, such as the US Food and Drug Administration (FDA), the European Medicines Agency (EMA), and the UK Medicine and Healthcare products Regulatory Agency (MHRA), committing to address regulatory issues unique to point-of-care manufacturing (EMA, 2022; FDA, 2022; MHRA, 2023). Specifically, discussions are being held regarding standardised terminologies, technical challenges, adaptation of Good Manufacturing Practices, and implementation of quality control measures.

The MHRA has notably proposed various alternative decentralised models of manufacturing, namely modular manufacturing (prefabricated self-contained manufacturing units), mobile manufacturing (micro-factories in specialised vehicles), and home-based or point-of-care manufacturing. Common to these alternative manufacturing options, often referred to as ‘distributed manufacturing’, is the shift of final dosage form assembly from centralised manufacturing sites to multiple delocalised locations. Consequently, pharmaceutical suppliers will depend on these decentralized satellite production facilities for the final customisation of pharmaceutical dosage forms (Öblom et al., 2019; Seoane-Viaño et al., 2021b). It is envisioned that drugs and excipients could be conventionally processed (e.g., blending, hot melt extrusion, and granulation) at a facility holding a manufacturing license under GMP procedures to produce 3D printing feedstock intermediate materials (“pharma-inks”) (i.e., granulates, filaments, powder blends). These pharma-inks will be quality controlled and delivered to the decentralized manufacturing sites to be transformed into administrable dosage forms (Beer et al., 2021; Seoane-Viaño et al., 2021b; Tracy et al., 2023).

Following manufacture, the quality of the printed dosage forms must be validated to safeguard patient safety and therapeutic efficacy (Beer et al., 2023; Quodbach et al., 2022). Conventionally, content uniformity and dose quantification in solid oral drug products are performed by chromatographic methods (e.g., high performance liquid chromatography (HPLC)), or by ultraviolet-visible spectrophotometry (UV–Vis) assays (Zeng et al., 2022)). However, these tests are performed on large batch samples and are inherently destructive, as the tablets must be disintegrated and dissolved prior to analysis (Vanhoorne and Vervaet, 2020). Moreover, such characterisation methods are costly and time consuming, making their inclusion in a clinical setting unfeasible (Deon et al., 2022). Therefore, to support the implementation of point-of-care manufacturing, non-destructive process analytical technologies (PAT) must be explored as an alternative for quality control (Deidda et al., 2019; Deon et al., 2022; Igne and Ciurczak, 2021).

Near-Infrared Spectroscopy (NIR) can be used as a PAT for quality control of 3DP drug products due to its non-destructive nature, high turnover rate, and comprehensive physicochemical insights (Talwar et al., 2022; Trenfield et al., 2023). Thus far, NIR technology has shown promising performance in quantifying drug content in 2D and 3D printed pharmaceuticals (Edinger et al., 2017; Pollard et al., 2023). However, to date, measurements have been conducted off-line, which involves removing the sample from the process flow prior to its analysis. To facilitate high throughput and streamlined production, all printlets in a single batch should instead be analysed immediately following production, using in-line technologies that have been integrated into the manufacture workflow (Harms et al., 2019; Melocchi et al., 2021; Sacré et al., 2021). In this way, printlets would not need to be diverted from the process stream, enabling real-time release testing at the point-of-care.

In light of the recent regulatory position supporting point-of-care manufacturing, this study aims to simulate and examine the feasibility of a plausible supply chain and decentralized manufacturing model for 3DP drug products. Herein, Losan Pharma GmbH (Germany) acted as the model pharmaceutical supplier, preparing pharma-inks in the form of efavirenz granulates and delivering it to a decentralized manufacturing site at the point-of-care (UCL School of Pharmacy). Efavirenz is a HIV type-1 nonnucleoside reverse transcriptase inhibitor widely used in combination with other drugs for the treatment of HIV. EFV is currently administered at a fixed dose of 600 mg once daily (Desta et al., 2019). However, efavirenz is metabolized by cytochrome P450 2B6 (CYP2B6), therefore patients with certain CYP2B6 genetic variants are more susceptible to adverse effects (Langmia et al., 2021). The large inter-patient variability in pharmacokinetics makes personalised dosing critical for these patients. The granulates were directly used to fabricate efavirenz printlets using a direct powder extrusion (DPE) 3D printer (Boniatti et al., 2021; Fanous et al., 2020; Ong et al., 2020; Pistone et al., 2023; Sánchez-Guirales et al., 2021). Subsequently, for the first time, an in-line NIR system was developed to perform in-process quality control of the printlets. Physicochemical characterisation and in vitro drug release studies were also performed to demonstrate the viability of the proposed model of manufacture.

## 2 Materials and methods

### 2.1 Materials

All granulated formulations were supplied by Losan Pharma GmbH (Germany). The drug efavirenz was also provided by Losan for quality control and analytical purposes, and was obtained from Zhejiang Jiangbei Pharmaceutical Co., Ltd. (Zhejiang, China). Kollidon VA64 was donated by BASF (Ludwigshafen, Germany). Mannitol 100 was purchased from Merck KGaA (Darmstadt, Germany). Magnesium Stearate was purchased from Peter Greven Nederland C.V. (Venlo, The Netherlands).

### 2.2 Preparation of granulated formulations

Efavirenz granulates were prepared according to the compositions shown in Table 1. All the granulates were prepared at Losan Pharma GmbH under GMP protocols conditions using qualified raw materials and valid, internal standard operating procedures for each manufacturing step. The applied dry granulation step by compression and subsequent sizing is crucial to acquire sufficient flowability for successful direct powder extrusion at the point of care. In detail, mannitol was sieved through 1.0 mm and blended for 10 min together with efavirenz, Kollidon VA64 and magnesium stearate using a Turbula mixer. The final blend was sieved again through 1.0 mm and compressed into round, biplane tablets (⌀ 25 mm, approx. 2 g) with a hardness of approx. 50–120 N. Milling was done on a Frewit MF-lab (oscillating milling machine, 70–100 osc./min). First, tablets were sieved/broken through 2.5 mm sieves. The sieve was subsequently changed, and the remaining granules were passed through a 0.71 mm sieve. Efavirenz granulates were kept in labelled plastic bottles and shipped to UCL School of Pharmacy for further processing into DPE 3D printed tablets.

**Table 1:** Formulations composition**.**

Formulation	Efavirenz (% w/w)	Kollidon VA64 (% w/w)	Mannitol 100 (% w/w)	Magnesium stearate (% w/w)	Printing temperature (°C)
Efa0	0	40	55	5	190
Efa5	5	40	50	5	180
Efa10	10	40	45	5	180
Efa15	15	40	40	5	180
Efa20	20	40	35	5	185
Efa25	25	40	30	5	180
Efa30	30	40	25	5	180
Efa35	35	40	20	5	180

### 2.3 3D printing process

**Fig. 1:**
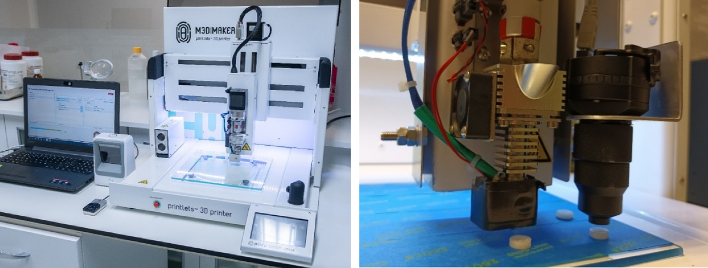
Pictures of the pharmaceutical 3D printer (left) and detail of the DPE printhead with the attached NIR probe (right).

The prepared granulated formulations were added to the hopper of the 3D printer DPE printhead (M3DIMAKER 1, FabRx, UK). The printer is specifically designed to prepare pharmaceutical products and it can incorporate different exchangeable tools. The selected tool was a direct single-screw powder extruder (FabRx, UK) with a nozzle diameter of 0.8 mm (Fig. 1). The design is based on a single-screw hot melt extrusion (HME); however, the rotation speed (and hence the extrusion) is controlled by the software of the 3D printer. Furthermore, the printhead moves in 3 dimensions to create the objects in a layer-by-layer fashion.

Autodesk 123D Design was used to design the templates of the printlets, exported as a stereolithography (.stl) file into 3D printer software (Repetier host v. 2.2.2, Germany). The. stl format contains only the object surface data, and all the other parameters need to be defined from the Repetier Host software to print the desired object. The selected 3D geometry was a round printlet (10 mm diameter × 3.6 mm height). The printer settings of the software were as follows: Infill 100%, feed rate 100 mm/s, flowrate: 100 mm/s, high resolution with brim, without raft and an extrusion temperature ranging from 180°C to 190°C depending on the formulation, speed while extruding (20 mm/s), speed while travelling (90 mm/s) and layer height (0.20 mm). The building plate was not heated during the printing process.

### 2.4 In-line NIR system

The NIR system was a portable MicroNIR 1700ES near infrared spectrometer (VIAVI, UK) equipped with 2 vacuum tungsten lamps and an InGaAs Photodioden Array detector for wavelengths between 950 and 1650 nm (= 10,526–6060 cm^−1^). Spectra were collected using a tablet probe with a 16 mm diameter collection optic attached to the MicroNIR device. A 99% spectralon reference standard (VIAVI, UK) was used for the acquisition of dark and reference spectra for instrument calibration prior to spectra acquisition.

To perform in-line data acquisition, the portable MicroNIR 1700ES with the tablet probe was attached to the moving part of the 3D printer, parallel to the printer printhead and perpendicular to the building plate. The MicroNIR™ spectrometer was controlled and data was collected using a specialised software (M3DIMAKER Studio – NIR section (FabRx, UK)). This software was especially developed to control the entire printing and quality control process. It provides instructions to the printer to move the printhead until the attached NIR tablet probe is placed above the printlet after each printlet is printed (Fig. 1). Then, the software automatically performs in-line data acquisition and collects reflectance spectra in triplicate. When the data acquisition is finished, the printhead moves to the next position to print the following printlet, and the process is repeated until the entire batch is printed.

### 2.5 Model development and multivariate data analysis

Printlets prepared with eight concentrations of efavirenz (*n* = 4) were used for model development (0%, 5%, 10%, 15%, 20%, 25%, 30%, 35%) (Table 1). Each printlet was analysed three times to avoid potential sampling errors. The final spectrum for each sample used to calculate efavirenz concentration was the average of the three spectra. Data pre-processing, multivariate data analysis, and modelling was performed using the M3DIMAKER Studio software – NIR section (FabRx, UK). Five printlet concentrations (*n* = 4) were selected for training the model (0%, 5%, 15%, 25%, 35%), and three concentrations (*n* = 4) were used for testing the model (10%, 20%, 30%) with the aim of measuring the performance of the model in a real scenario on unseen data. Partial least squares (PLS) regression was performed on the datasets to build calibration models. A 10-fold cross validation with 3 repeats was applied to the models.

PLS model graph of NIR predicted vs. HPLC determined efavirenz content was created using GraphPad Prism 8 (San Diego, California, US) using the data extracted from the M3DIMAKER Studio – NIR section software. Following NIR analysis, each individual printlet was quantitatively analysed for drug content via HPLC following the methodology described in Section 2.6.

### 2.6 Determination of drug loading

All printlets used for NIR analysis were individually placed in volumetric flasks with methanol:water 1:1 solution, followed by magnetic stirring until complete dissolution (*n* = 4). Samples of the solutions were then filtered through 0.45 μm PVDF filters (Millipore Ltd., Ireland) and the concentration of drug determined with high-performance liquid chromatography (Agilent 1290 Infinity UHPLC, Agilent Technologies, UK). The stationary phase was an Aquity BEH 1.7 μm C18 column, 100 × 2.1 mm (Waters GmbH, Eschborn, Germany), and the mobile phase was a combination of 70% phosphate buffer pH 3.0 and 30% methanol pumped at a flow rate of 0.3 mL/min. The injection volume was 10 μL, and column temperature was set to 25°C with UV-wavelength of 247 nm. The elution time of efavirenz was approximately 6 min. Data were processed using Chromeleion software (Thermo Scientific, MA, US).

### 2.7 Characterisation of the printlets

#### 2.7.1 Determination of printlet morphology

The dimensions (diameter and thickness) of the printlets were measured using a digital Vernier calliper. A Sartorius Entris 124-1S analytical balance was used to determine the mass of each printlet.

#### 2.7.2 Determination of printlet strength

The breaking force of the printlets was measured using a traditional tablet hardness tester TBH 200 (Erweka GmbH, Heusenstamm, Germany), whereby an increasing force is applied perpendicular to the tablet axis to opposite sides of a tablet until the printlet fractures (*n* = 3).

#### 2.7.3 Scanning electronic microscopy (SEM)

SEM images were obtained using a scanning electron microscope Phenom Pro (Phenom-World BV, Eindhoven, Netherland) with an acceleration voltage of 10 kV. Small sections of the 3D printed discs (8 mm diameter × 1 mm height) were fixed on 25 mm aluminium stubs using self-adhesive carbon discs and coated with gold for 60 s using a rotatory pumped coater (Q150R S plus, Quorum, UK).

#### 2.7.4 X-ray powder diffraction (XRPD)

3D printed discs (23 mm diameter × 1 mm height) were analysed alongside efavirenz and different powder blends. X-ray powder diffraction patterns were obtained using a Rigaku MiniFlex 600 (Rigaku, USA) equipped with a Cu K α X-ray source (λ = 1.5418 Å). The angular range of data acquisition was 3–60° 2 θ with a stepwise size of 0.02° at a speed of 5°/min. The intensity and voltage applied were 15 mA and 40 kV, respectively.

#### 2.7.5 Thermal analysis

Differential scanning calorimetry (DSC) was used to characterise efavirenz and different powder blends and printlets. DSC measurements were performed with a Q2000 DSC (TA Instruments, Waters, LLC, U.S.A.) at a heating rate of 10°C/min. Calibration for cell constant and enthalpy was performed with indium (Tm = 156.6°C, ΔHf = 28.71 J/g) according to the manufacturer's instructions. Nitrogen was used as a purge gas with a flow rate of 50 mL/min for all the experiments. Data were collected with TA Advantage software for Q series (version 2.8.394). Melting temperatures are reported as an extrapolated onset unless otherwise stated. TA aluminium pans and lids (Tzero) were used with an average sample mass of 3–5 mg.

#### 2.7.6 In vitro drug release study

In vitro drug release profiles were obtained using a USP-II apparatus (Model AT 7 Smart, Sotax, US). The printlets were placed in water with 2% SDS as indicated in the USP monograph for efavirenz tablets (500 or 900 mL depending on drug loading, 500 mL for Efa5 and Efa10 and 900 mL for the other concentrations) under constant paddle stirring (100 rpm) at 37°C. The percentage of drug released from the printlets was determined using an in-line UV spectrophotometer at 292 nm. Data were processed using WinSotax software (Sotax, US). Tests were conducted in quadruplicate.

## 3 Results and discussion

**Fig. 2:**
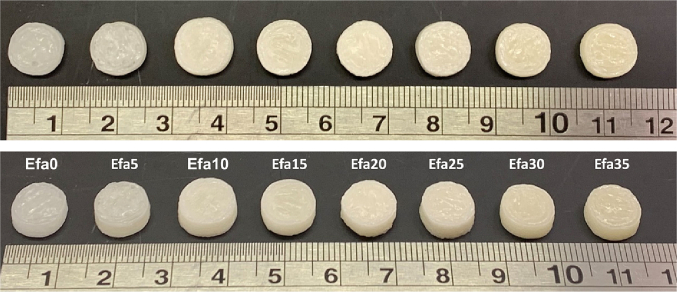
Images of the different 3D printed tablets with varying concentrations (from 0 to 35% of drug loading) of the drug efavirenz. Scale shown in cm.

All the granulated formulations were found suitable for DPE 3D printing (Fig. 2). All the printlets presented a cylindrical shape and smooth surface with good adhesion between the printed layers. A change in colour can be appreciated with increasing concentrations of the drug.

Powder formulations with intermediate concentrations of efavirenz (10–25%*w*/w) had better extrusion properties. Different relative amounts of efavirenz in the powder formulations resulted in different flow properties, which influenced the amount of melted polymer blend that was deposited by the printhead. This resulted in the observed differences in mass between the printlets derived from the different formulations (Table 2), ranging from 266 mg to 371 mg even though the same CAD file was used. Printlets derived from 10 and 15%w/w efavirenz formulations had the largest mass. Nonetheless, there is minimal intra-batch variability and the drug loading of the obtained printlets agreed with the theoretical values.

**Table 2:** Physical properties and drug loading of the Printlets (*n* = 4).

Formulation	Weight ± SD (mg)	Diameter ± SD (mm)	Thickness ± SD (mm)	Breaking Force ± SD (N)	Drug loading ± SD (%)
Efa0	266.9 ± 0.01	9.69 ± 0.02	3.24 ± 0.07	102.5 ± 0.7	0
Efa5	303.4 ± 0.01	9.91 ± 0.25	3.34 ± 0.05	281.3 ± 3.05	5.16 ± 0.05
Efa10	371.5 ± 0.01	10.70 ± 0.09	3.60 ± 0.04	283 ± 1	10.16 ± 0.03
Efa15	338.6 ± 0.01	10.33 ± 0.27	3.36 ± 0.04	177 ± 31.11	15.20 ± 0.03
Efa20	334 ± 0.01	10.10 ± 0.20	3.53 ± 0.16	103 ± 1.41	19.94 ± 0.08
Efa25	327.4 ± 0.01	10.17 ± 0.19	3.40 ± 0.01	185 ± 12.72	24.69 ± 0.07
Efa30	335.4 ± 0.01	9.71 ± 0.58	2.97 ± 0.24	139.5 ± 12.02	30.39 ± 0.09
Efa35	327.4 ± 0.01	10.17 ± 0.04	3.39 ± 0.03	81.5 ± 9.19	35.55 ± 0.15

Efavirenz printlets also displayed good uniformity in physical dimensions (Table 2). The diameter of the tablets was between 9.69 ± 0.02 and 10.70 ± 0.09 mm with a thickness between 2.97 ± 0.54 and 3.60 ± 0.04 mm, values close to the theoretical size (10 mm diameter x 3.6 mm height). All the printlets were mechanically strong, with breaking force values ranged between 81.5 ± 9.19 and 283 ± 1 N, which are comparable to those previously reported from tablets prepared using DPE.

**Fig. 3:**
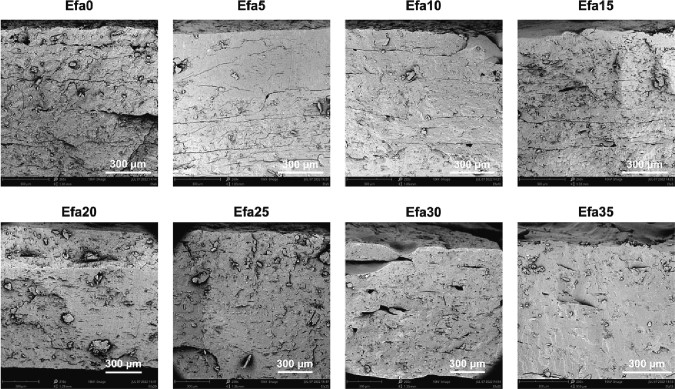
SEM images of cross-sections of different printlets. The scale bar is equivalent to 300 μm.

SEM images of the cross-section of the printlets show a dense but slightly rough inner matrix (Fig. 3). Traces of the layers formed by the deposition of material during printing can be seen in Efa0 to Efa15, and the other formulations displaying a more homogeneous inner matrix. No crystalline drug deposition was observed in any of the formulations. Some small holes can be observed in some of the images, probably formed by trapped air bubbles during the extrusion process.

**Fig. 4:**
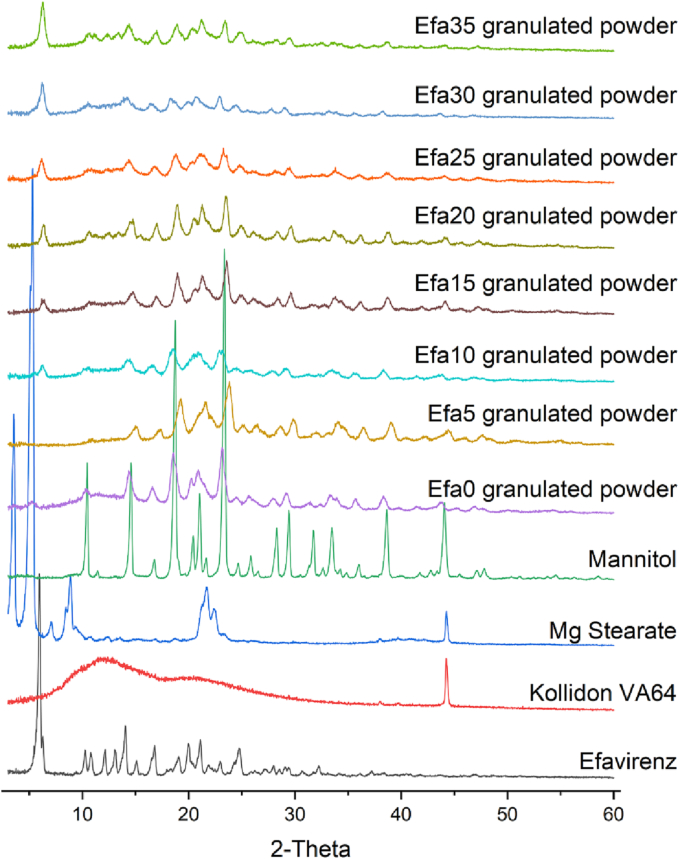
X-ray powder diffractograms of efavirenz and different granulates mixtures.

**Fig. 5:**
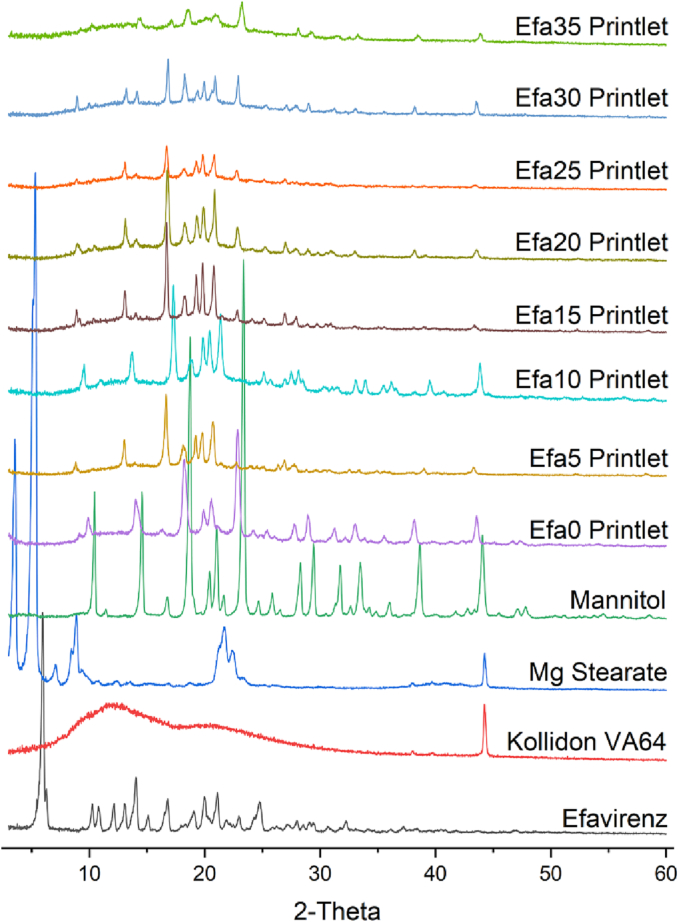
X-ray powder diffractograms of efavirenz and different printlets.

XRPD and DSC analysis was performed to investigate the physical state of efavirenz in the granulates of the formulations and in the printlets. X-ray powder diffractograms showed the characteristic peaks of efavirenz at 5.9°, 12.1°, 14.0°, and 16.8° 2θ (Fig. 4). Specifically, the diffractogram of efavirenz observed from this study corresponds well with that of polymorph I of efavirenz (Fandaruff et al., 2014). The peak at 5.9° was clearly observed in all the granulated powder mixtures, indicating the crystalline form of efavirenz prior printing. However, absence of this signal after the printing process (Fig. 5) suggested efavirenz existed in amorphous form within the printlets.

**Fig. 6:**
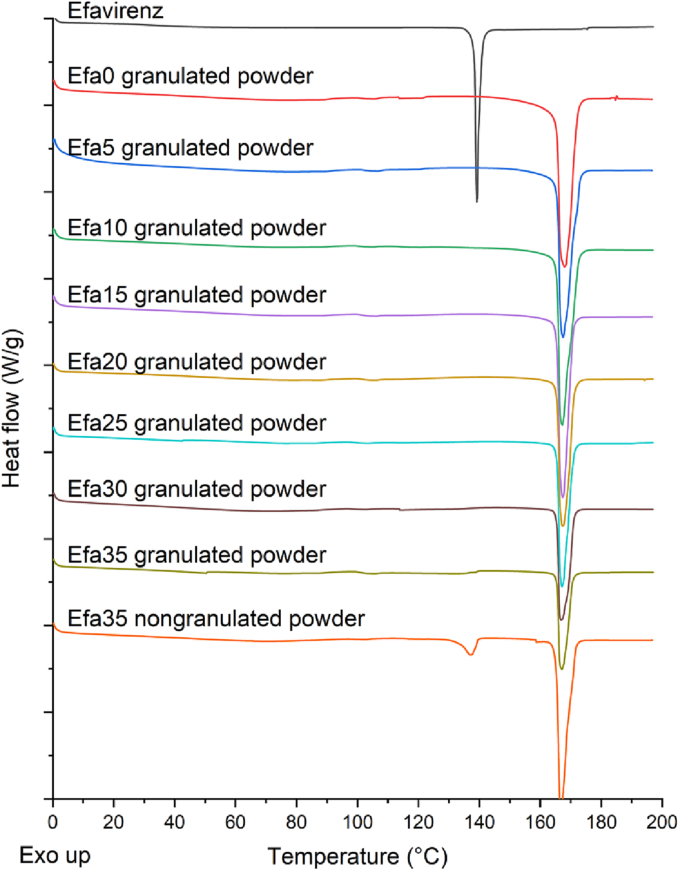
DSC thermograms of efavirenz and different granulates and Efa35 powder mixtures.

On the other hand, DSC results showed that efavirenz pure powder exhibited a melting endotherm at 139.2°C (Sathigari et al., 2009) (Fig. 6). This corresponds to the experimental melting point of efavirenz polymorph I observed by Fandaruff et al., thereby supporting the observation that polymorph I is the predominant polymorphic form of efavirenz present in the samples (Fandaruff et al., 2014). The same study had also found that polymorphs I and II of efavirenz are enantiotropically related with an isoenergetic point between 35 and 40°C (Fandaruff et al., 2014). However, no endotherms were observed at this temperature range in the thermogram of efavirenz, suggesting that no enantiotropic conversion occurred. The endothermic peak at around 168°C in the Efa0 granulated powder corresponds to the melting point of mannitol (Allahham et al., 2020), which can be seen in all the powder mixtures. Interestingly, the endothermic peak corresponding to the melting of efavirenz was not observed in any of the granulated powder mixtures. This is possibly due to the dry granulation process where the efavirenz drug particles were aggregated with other excipients under high pressure. During the DSC experiment, efavirenz dissolved in the molten Kollidon VA64 whose Tg is at around 101°C (Allahham et al., 2020) upon slow heating (10°C/min) hence the melting signal was masked. To investigate the hypothesis, Efa35 nongranulated powder was prepared and DSC was performed using the same condition. The data clearly showed a small melting peak of efavirenz at around 139°C, confirming the hypothesis.

**Fig. 7:**
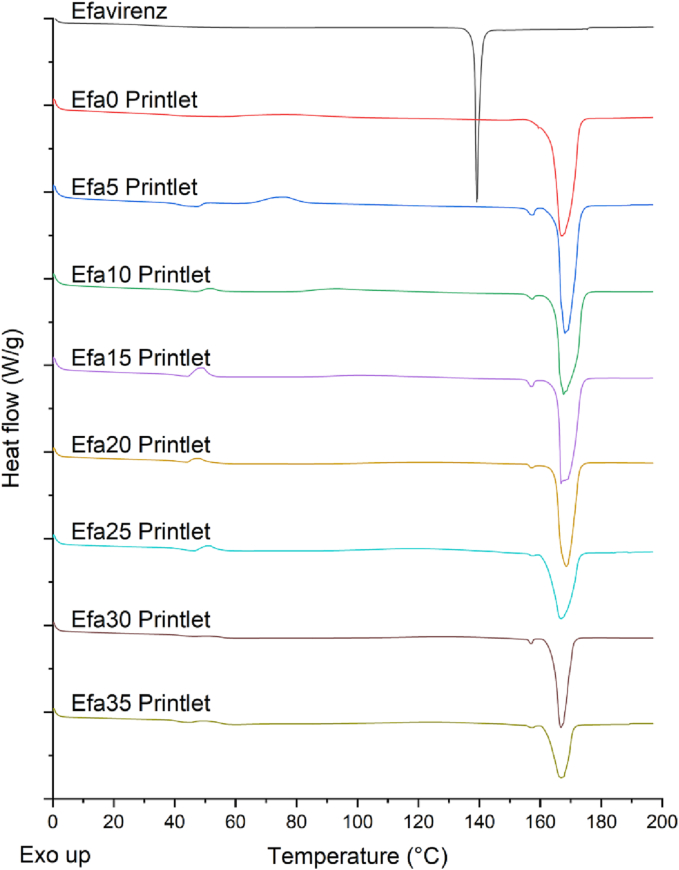
DSC thermograms of efavirenz and different printlets.

After DPE 3D printing, DSC data showed an endotherm at 168°C in all the Printlets which refers to the melting of mannitol (Fig. 7). The absence of the endothermic peak at 139°C indicated the amorphous form of efavirenz within the Printlets, corroborating with the XRPD results.

NIR spectroscopy was used for the quantification of the drug in the Printlets. Following NIR analysis, each individual printlet was quantitatively analysed for drug content using HPLC. The observed drug loading values for different concentrations were close to the theoretical values (Table 2). In-line NIR spectra were measured during the production process right after each tablet was printed. To do so, it was necessary to develop a printhead with an accessory to hold the NIR tablet probe so that the printhead nozzle and the tablet probe were located the same distance from the build plate. The specialised software used to control the NIR and the 3D printer (M3DIMAKER Studio – NIR section) begins by placing the NIR tablet probe on top of a reference standard for acquisition of dark and reference spectra for instrument calibration. After the first tablet is printed, the software sends instructions to move the printhead until the NIR probe is placed on top of the tablet. Then, the software triggers a signal to collect reflectance spectra and also instructs the printer to wait until the spectra have been collected in triplicate. The collected data are then analysed as the printhead moves to the next location to print the next tablet. This process is repeated until the entire batch is printed. NIR quantification could be either performed during the printing process after each printlet or after the entire batch is printed. Doing the quantification after the entire batch speeds up the process.

**Fig. 8:**
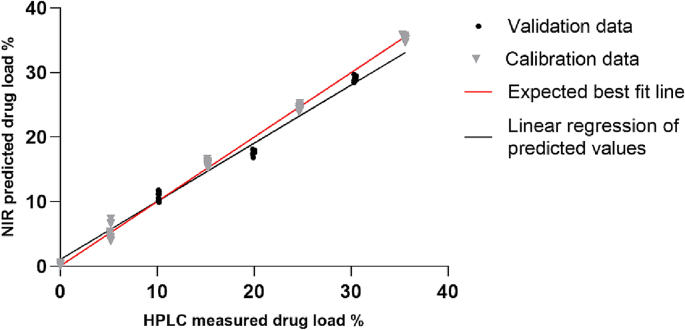
PLS model of NIR predicted vs. HPLC determined efavirenz content. Grey points are calibration (5 concentrations) and black are validation (3 concentrations).

To allow the real-time data analysis, a multivariate calibration model was also created using different pre-processing techniques and included in the software. To create a reliable calibration model, data pre-treatment is necessary to remove unwanted variations, such as experimental artifacts, and to improve accuracy of quantification (Rinnan et al., 2009). In this study, 6 PLS models (Table 3) were developed with 3 different pre-treatment filters (Standard Normal Variate (SNV), Multiplicative Scatter Correction (MSC) and Savitzky-Golay smoothing) and their combinations were applied to the spectra in a wavelength range between 950 and 1650 nm. A 10-fold cross validation with 3 repeats was performed to determine the optimal number of latent variables (LVs) in the PLS model. The model with the higher linearity (R^2^ test = 0.9833) and the lowest root mean square error (RMSE test = 1.0662%) was selected, which corresponds to model number 4 (Table 3) (Fig. 8). The selected model used 2nd derivative (Savitzky–Golay with a filter width of 25 and a 2nd polynomial) pre-processing technique.

**Table 3:** Comparison of different spectral pre-processing methods used to develop the calibration models. SNV: standard normal variant, MSC: multiplicative scatter correction, SG: second derivative spectra (Sativzky-Golay method).

Model number	Data pre-treatment	Latent variables	R^2^ training	RMSE (%) training	R^2^ test	RMSE (%) test
1	None	4	0.9974	0.6538	0.9115	2.4569
2	MSC	4	0.9942	0.9802	0.8708	2.9692
3	SNV	4	0.9994	0.2994	0.9643	1.5610
4	SG	4	0.9967	0.7397	0.9833	1.0662
5	MSC + SG	4	0.9956	0.8523	0.9360	2.0891
6	SNV + SG	4	0.9977	0.6145	0.9516	1.8174

**Fig. 9:**
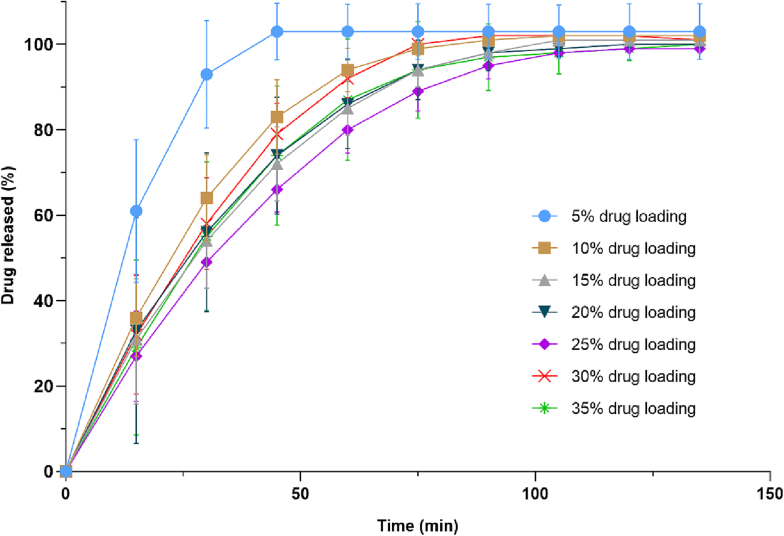
Cumulative drug release profiles of efavirenz printlets.

Dissolution profiles showed that more than 80% of efavirenz was released in the first 60 min for all concentrations, reaching 100% of drug release within 120 min (Fig. 9). The Efa5 Printlets showed a faster release rate, possibly due to inconsistent extrusion of material during the printing process. This could have been caused by lack of plasticity of the extruded material due to its lower content in drug, leading to variable pressures within the printhead. Consequently, the small gaps within Efa5 Printlets increased the surface-area-to-volume ratio, accelerating drug release.

This study has successfully demonstrated the feasibility of a model of point-of-care manufacturing wherein pharma-inks (akin to bioinks in bioprinting) is prepared by a pharmaceutical industry partner under GMP, delivered to and fabricated at the satellite manufacturing site, and quality controlled with an in-process PAT tool (NIR spectroscopy). While the pharma-inks demonstrated in this study are solid, ink may be either solid or liquid. Therefore, pharma-inks encompasses feedstock intermediate for any pharmaceutical 3D printing technique, such as liquid pharma-inks in stereolithography and solid pharma-inks in direct powder extrusion. The 3D printing process and the acquisition of the printlets' NIR spectra were both controlled by a unified software, facilitating accelerated throughput and batch release. Integration of other PAT tools could allow even more comprehensive and robust analysis of printed drug products to ensure they possess the appropriate final critical quality attributes (CQAs). For example, a pressure sensor was previously integrated into a semi-solid extrusion 3D printer to monitor the printing process while characterizing the rheological properties of inks (Díaz-Torres et al., 2022).

Apart from integrating numerous PAT tools within a pharmaceutical 3D printer for multi-modal analysis, future work could include investigating the selectivity of these QC measures. There are inadvertently multiple points along an international supply chain wherein the pharma-inks cargo may be intercepted and replaced with falsified or counterfeit products. Therefore, highly compound-selective in-line PAT tools may serve as a countermeasure against illegal interception of pharma-inks, further safeguarding patient safety.

## 4 Conclusion

In this study, the point-of-care manufacturing of personalised printlets derived from pharma-inks prepared and delivered by a pharmaceutical company was simulated. Specifically, efavirenz-loaded printlets were successfully fabricated using DPE 3D printing and their drug content was quantified using an in-line NIR system. The developed NIR calibration models demonstrated excellent linearity (R^2^ = 0.9833) and accuracy (RMSE = 1.0662). The developed in-line dose verification system enables QC analysis to be performed immediately after fabrication, facilitating high throughput and real-time batch release in clinics in the future. Integration of other PAT tools in future work could enable more comprehensive multi-modal analysis to support the deployment of 3D printers at the point-of-care.

## CRediT authorship contribution statement

**Iria Seoane-Viaño:** Data curation, Formal analysis, Investigation, Methodology, Writing – original draft, Writing – review & editing. **Xiaoyan Xu:** Data curation, Formal analysis, Investigation, Methodology, Writing – original draft, Writing – review & editing. **Jun Jie Ong:** Formal analysis, Investigation, Methodology, Writing – review & editing. **Ahmed Teyeb:** Formal analysis, Methodology, Writing – review & editing. **Simon Gaisford:** Supervision, Resources, Writing – review & editing. **André Campos-Álvarez:** Data curation, Formal analysis, Investigation, Methodology. **Carmen Marcuta:** Data curation, Formal analysis, Investigation, Methodology, Writing – review & editing. **Lilia Kraschew:** Data curation, Formal analysis, Investigation, Methodology, Writing – review & editing. **Wolfgang Mohr:** Data curation, Formal analysis, Investigation, Methodology, Writing – review & editing. **Abdul W. Basit:** Conceptualization, Methodology, Project administration, Resources, Supervision, Writing – review & editing. **Alvaro Goyanes:** Conceptualization, Methodology, Project administration, Resources, Supervision, Writing – review & editing.

## Declaration of Competing Interest

The authors declare that they have no known competing financial interests or personal relationships that could have appeared to influence the work reported in this paper.

## Data Availability

Data will be made available on request.
